# Dose-Response Relationship of Uric Acid With Fasting Glucose, Insulin, and Insulin Resistance in a United States Cohort of 5,148 Non-diabetic People

**DOI:** 10.3389/fmed.2022.905085

**Published:** 2022-06-09

**Authors:** Yingdong Han, Xinxin Han, Yue Yin, Yu Cao, Hong Di, Juan Wu, Yun Zhang, Xuejun Zeng

**Affiliations:** Department of Family Medicine and Division of General Internal Medicine, Department of Medicine, Peking Union Medical College Hospital, Chinese Academy of Medical Sciences, State Key Laboratory of Complex Severe and Rare Diseases (Peking Union Medical College Hospital), Beijing, China

**Keywords:** uric acid, insulin resistance, fasting glucose, hyperinsulinemia, menopausal status, NHANES

## Abstract

**Background:**

There is a limited number of studies on the dose-response relationship between serum uric acid and impaired glucose metabolism in people without diabetes, and no large-scale research exploring the relationship in women without diabetes is based on menopausal status. Consequently, the present study aimed to investigate the above relationship in United States adults without diabetes.

**Materials and Methods:**

Data from 2,498 men and 2,650 women aged ≥20 years were obtained from the National Health and Nutrition Examination Survey 2011–2016 conducted in the United States. Binary logistic regression analysis was applied to evaluate the association between uric acid and impaired glucose metabolism. Restricted cubic spline analysis, sensitivity analysis, and stratified analysis by menopausal status were performed to explore the above relationships.

**Results:**

A positive correlation was found between uric acid and the risk of insulin resistance in all participants (*P* < 0.05). In binary logistic regression analysis, after adjusting for confounding factors, compared with the lowest quartile of uric acid, the odds ratio (95% confidence intervals) of insulin resistance in the highest quartile was 1.9 (1.1–3.1) and 2.2 (1.2–4.3) in men and women, respectively. A significant positive relationship was also observed between uric acid and impaired fasting glucose and hyperinsulinemia in women, while in men, uric acid was positively associated with the risk of hyperinsulinemia but not impaired fasting glucose. Restricted cubic spline showed that the odds ratios of insulin resistance and hyperinsulinemia increased with elevating uric acid levels in both men and women. When stratified by menopause, the association remained significant in pre-menopausal women aged ≥20, but insignificant in post-menopausal women.

**Conclusion:**

Uric acid was positively associated with the risk of impaired glucose metabolism in a cohort of United States adults, and uric acid increased the risk of insulin resistance in pre-menopausal, but not in post-menopausal women.

## Introduction

Uric acid (UA) is the end product of purine nucleotide metabolism. Approximately, two-third of UA is excreted by the kidneys and one-third by the gastrointestinal tract, and its synthesis and excretion are balanced under physiological conditions (1, 2). Hyperuricemia is the precursor of gout that can occur due to overproduction or underexcretion of serum uric acid (SUA). In addition to gout, numerous research studies have suggested the clinical significance of SUA beyond the rheumatologic field (3), such as impaired glucose metabolism, abnormal lipid metabolism, and higher prevalence of obesity, hypertension, and metabolic syndrome (4–10). Previous studies have also suggested a bidirectional causal effect between SUA and insulin resistance (IR) (11). SUA was found to affect the function of islet β cells and was inversely associated with insulin sensitivity (11, 12).

Insulin exerts its physiological function through a cascade of signaling transduction by enhancing glucose disposal in insulin-sensitive tissues. IR is defined as the inability of insulin to optimally stimulate the transport of glucose into cells (13). It is also considered a hallmark of metabolic syndrome and an important pathogenic factor of type 2 diabetes mellitus (11, 14). Impaired fasting glucose and compensatory hyperinsulinemia are accompanying characteristics of IR. Moreover, IR is closely associated with dyslipidemia, coronary artery disease, and hypertension (15), while abdominal obesity, acanthosis nigricans, and acne are considered their major clinical features (16). In addition, restricting calorie intake and exercising are associated with improved insulin sensitivity, while aging, smoking, and inactive physical activity are positively associated with the risk of IR (16).

Accumulating evidence demonstrated that SUA is associated with impaired glucose metabolism; however, these results appear to be inconsistent. Some clinical studies found that elevated SUA was associated with a higher risk of IR, hyperinsulinemia, fasting plasma glucose, and HbA1c level (17, 18). However, in their study on 605 newly diagnosed type 2 diabetes patients, Ma et al. found that SUA was inversely correlated with HbA1c in the high insulin group, while no associations were found in the low insulin group (19).

Previous studies on the association between SUA and glucose metabolism mostly included prediabetic or diabetic patients. Some limited small-scale studies investigated the above association in United States adults without diabetes, and no large-scale research explored the above relationship in women without diabetes based on menopausal status. Therefore, the present study included a nationally representative cohort of United States adults without diabetes whose data were obtained from the National Health and Nutrition Examination Survey (NHANES) 2011–2016 to explore the dose-response relationship between SUA and IR, impaired fasting glucose, and hyperinsulinemia. We performed restricted cubic spline analysis and stratified analysis by menopausal status to explore the above relationship with a large-scale nationwide sample for the first time to provide a valuable reference for the impaired glucose metabolism and further study of IR in women with different menopausal statuses.

## Materials and Methods

### Data Source and Study Population

The NHANES, which collects the health and nutritional information of the United States population every 2 years, is conducted by the Centers for Disease Control and Prevention of America. Through a multistage, stratified sampling design, NHANES included a representative sample of non-institutionalized United States civilians. After a detailed in-home interview, a physical examination and blood and urine specimens were obtained at specially equipped mobile examination centers (20). Written informed consent was obtained before the interview, and examination stages from all participants and all data were de-identified by the National Center for Health Statistics before being made publicly available.

**FIGURE 1:**
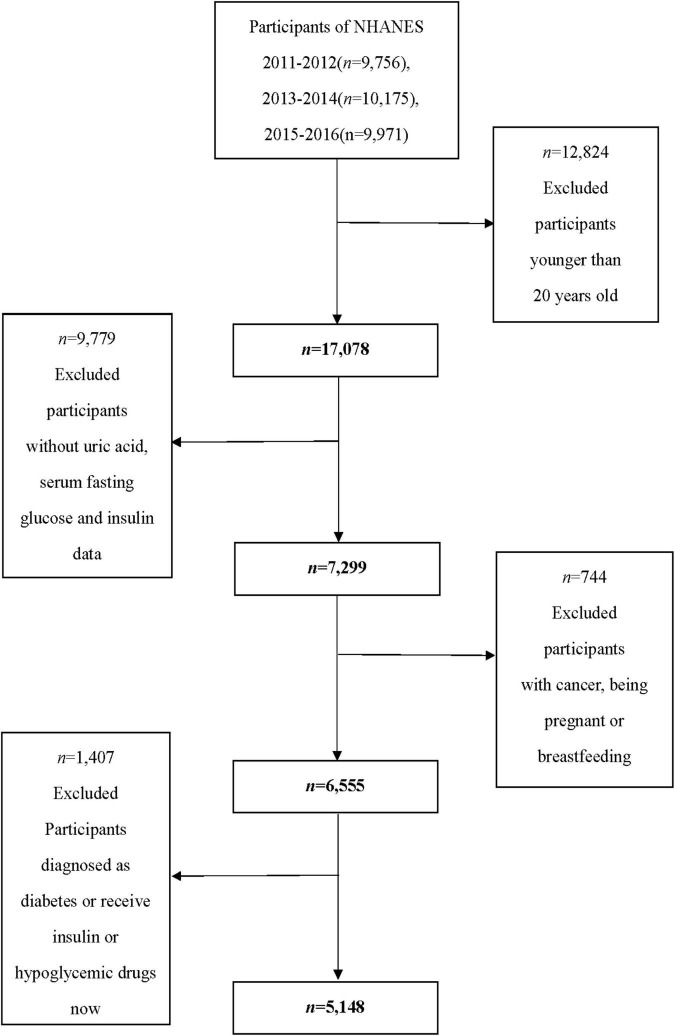
Flow chart of the screening process for the selection of eligible participants.

A total of 29,902 adult participants were enrolled in the NHANES between 2011 and 2016. Exclusion criteria were the following: (1) age <20 years (*n* = 12,824); (2) participants whose SUA data (*n* = 1,718) and serum fasting glucose and insulin data (*n* = 8,061) were missing; (3) self-reported cancer or malignancy (*n* = 670); (4) being pregnant or breastfeeding (*n* = 74); (5) self-reported diabetes, fasting glucose ≥ 7.0 mmol/L or 2-h glucose of oral glucose tolerance test ≥ 11.1 mmol/L, HbA1C ≥ 6.5%, and receive insulin or hypoglycemic drugs now (*n* = 1,407). Eventually, 2,498 men and 2,650 women were included in our study. The flow chart of the screening process is shown in Figure 1.

### Study Variables

Variables in this study included age, race (Mexican American, Other Hispanic, Non-Hispanic White, Non-Hispanic Black, and Other race), body mass index (BMI) (normal: <25 kg/m^2^; overweight: 25–30 kg/m^2^; and obesity: ≥ 30 kg/m^2^) (21), education level (Less than 9th grade, 9–11th grade, High school graduate, College degree, and College graduate or above), waist circumference, and estimation of the glomerular filtration rate (eGFR) [eGFR = 175 × standardized Scr^−1.154^ × age^−0.203^ × 1.212 (if black) × 0.742 (if women)](22). The poverty income ratio (PIR) was used to define income, categorized as less than 0.99 and 1 or more; a PIR < 1.0 represented a person living under the poverty line. Total cholesterol, triglyceride, smoking status (smoked at least 100 cigarettes in life or not), and drinking status (had at least 12 alcohol drinks/1 year) were also included in the present study. A history of hypertension is defined as the self-reported diagnosis of hypertension by a physician. Urate-lowering therapy was also adjusted in our study. NHANES questions, *“In the past 30 days, have you used or taken medication for which a prescription is needed?”* and *“Generic drug name,”* revealed that 10 women and 29 men were using uric lowering therapy (allopurinol, febuxostat, and probenecid).

### Definitions

The homeostasis model assessment of IR (HOMA-IR) is a common indirect index for the assessment of IR. HOMA-IR = [fasting insulin (mU/L) × fasting glucose (mmol/L)]/22.5 (23). IR was defined as HOMA-IR ≥ 75th percentile (HOMA-IR ≥ 3.55 for men and ≥ 3.45 for women) by gender in our study population (24). Impaired fasting glucose (IFG) was defined as 6.1 mmol/L ≤ fasting plasma glucose < 7.0 mmol/L (25). Hyperinsulinemia was defined as serum insulin ≥ 75th percentile by gender (Insulin ≥ 14.15 for men and ≥ 14.03 for women) (26). Menopause was defined by answering “no” to the question “Have you had at least one menstrual period in the past 12 months?” or age ≥ 55 (27).

### Statistical Analysis

All statistical analyses were conducted with SPSS 23, STATA 15.0, and R 4.1.0. The normality of the continuous variable was tested with the Kolmogorov–Smirnov normality test. Normally distributed variables were described with mean ± SD, while non-normally distributed continuous variables were described with median (interquartile range). The median values among different SUA groups were compared with the Kruskal–Wallis test. The percentages of categorical variables among different SUA groups were compared with the Chi-square test. The Bonferroni test was used for the intergroup comparison. As the SUA level substantially differed between men and women, the analyses were performed separately by gender. SUA levels in binary logistic regression analyses were modeled in quartiles: Men groups: Q1 (uric acid ≤ 309.30 μmol/L), Q2 (309.30 < uric acid ≤ 356.90 μmol/L), Q3 (356.90 < uric acid ≤ 404.50 μmol/L), and Q4 (uric acid > 404.50 μmol/L); Women groups: Q1 (uric acid ≤ 232.00 μmol/L), Q2 (232.00 < uric acid ≤ 273.60 μmol/L), Q3 (273.60 < uric acid ≤ 321.20 μmol/L), and Q4 (uric acid > 321.20 μmol/L). The lowest quartile (Q1) group was used as the reference group. Binary logistic regression analysis examined the association between SUA and IR, impaired fasting glucose, and hyperinsulinemia. We compared the clinical characteristics of the study population with or without IR, and the covariates with significant differences between the two groups were included in the full-adjusted Model 2. Finally, the race was adjusted in Model 1, and Model 2 was additionally adjusted for BMI, waist circumference, drinking status, education level, hypertension, total cholesterol, triglyceride, and urate-lowering therapy.

Logistic regression analysis was performed to calculate the odds ratio (OR) values per SD increase in SUA. We also performed linear regression analysis to assess the association between SUA and HOMA-IR. Stratified analysis and trend tests were used for female participants based on menopausal status, as the SUA levels increased substantially after menopause (28). Participants with no available information about menopause were excluded from the stratified analysis. In addition to menopause, stratified analysis based on BMI (normal: < 25 kg/m^2^; overweight: between 25 and 30 kg/m^2^; and obesity: ≥ 30 kg/m^2^), waist circumference (cutoff value for American: 102 cm for men and 88 cm for women), serum triglyceride (cutoff value: 1.7 mmol/L), total cholesterol (cutoff value: 5.2 mmol/L) (29), drinking status (Yes/No), education levels (college and above/high school and below), and hypertension (Yes/No) were also performed. Subsequently, a sensitivity analysis was performed by excluding participants without metabolic syndrome to minimize the influence of metabolic syndrome. Restricted cubic spline analysis with 3 knots of the SUA levels was used to characterize the dose-response relationship in the logistic regression Model 2. The function “lrm” and function “rcs” in the “rms” package of R 4.1.0. were used to fit the independent and dependent variables. The reference values were automatically determined by “rcs” function. The function “predict” was used to calculate the ORs (95% CIs) and reference point (OR = 1.0) of SUA. A two-sided *p* < 0.05 was considered statistically significant.

## Results

A total of 5,148 participants (2,498 men, 2,650 women) were included in the study and categorized into five racial groups: Mexican American (*n* = 682), Other Hispanic (*n* = 567), Non-Hispanic White (*n* = 2,000), Non-Hispanic Black (*n* = 1,020), and other races (*n* = 879). The mean age was 45.21 ± 16.53 years, and the mean SUA was 320.93 ± 81.80 μmol/L. There were 20.1% of male participants and 12.9% of female participants who met the hyperuricemia criteria; 11.7% of all participants were with an SUA concentration ≥420 μmol/L. The clinical characteristics of individuals from different SUA groups are shown in Tables 1, 2. A greater proportion of participants with IR or hypertension belonged to the highest quartile of SUA level; with the increasing of SUA quartiles, the median of eGFR gradually declined, while the median of BMI, waist circumference, cholesterol, triglyceride, serum insulin, creatinine, and HOMA-IR gradually increased in both men and women. Among men, the proportion of those living under the poverty line gradually declined with the increase of SUA quartiles. The clinical characteristics of individuals with or without IR are shown in Table 3. The waist circumference, BMI, triglyceride, total cholesterol level, glycohemoglobin, and prevalence of hypertension were significantly higher in the IR group.

**TABLE 1:** Clinical characteristics of the study population disaggregated by quartiles of serum uric acid level.

Serum uric acid quartile	Q1	Q2	Q3	Q4	*P*-value
Number of subjects	635	685	567	611	
Age (year)^b^	44 (27)	42 (26.5)	42 (24)	42 (28)	0.221
Race (%)^a^					0.103
Mexican American	95 (15.0)	93 (13.6)	88 (15.5)	62 (10.1)	
Other Hispanic	70 (11.0)	85 (12.4)	65 (11.5)	55 (9.0)	
Non-Hispanic White	240 (37.8)	266 (38.8)	222 (39.2)	246 (40.3)	
Non-Hispanic Black	127 (20.0)	116 (16.9)	98 (17.3)	129 (21.1)	
Other race	103 (16.2)	125 (18.2)	94 (16.6)	119 (19.5)	
Education level (%) ^a^					< 0.01
Less than 9th grade	71 (11.2)	56 (8.2)	48 (8.5)	30 (4.9)	
9–11th grade	92 (14.5)	100 (14.6)	87 (15.3)	92 (15.1)	
High school graduate	160 (25.2)	139 (20.3)	141 (24.9)	143 (23.4)	
College or AA degree	159 (25.0)	211 (30.8)	157 (27.7)	165 (27.0)	
College graduate or above	153 (24.1)	179 (26.1)	134 (23.6)	181 (29.6)	
Waist circumference (cm)^b^	91.6 (17.8)	95.5 (17.1)	99.3 (15.8)	102.2 (20.5)	< 0.01
Body mass index (kg/m^2^)^b^	24.9 (5.75)	26.5 (6.03)	28.2 (6.20)	29.3 (7.6)	< 0.01
Cholesterol (mmol/L)^b^	4.65 (1.37)	4.81 (1.34)	4.97 (1.30)	5.04 (1.35)	< 0.01
Triglyceride (mmol/L)^b^	0.96 (0.70)	1.12 (0.79)	1.28 (1.01)	1.48 (1.24)	< 0.01
Creatinine (μmol/L)^b^	79.6 (19.5)	83.1 (17.7)	84.9 (19.5)	88.4 (20.3)	< 0.01
eGFR (mL/min per 1⋅73m^2^)^b^	97.27 (27.68)	92.33 (24.04)	90.66 (27.90)	84.91 (25.84)	< 0.01
Insulin (mU/L)^b^	7.10 (6.04)	7.94 (7.68)	10.03 (9.23)	11.92 (12.16)	< 0.01
Fasting glucose (mmol/L)^b^	5.49 (0.67)	5.49 (0.61)	5.50 (0.67)	5.61 (0.67)	< 0.01
HOMA-IR^b^	1.74 (1.56)	1.96 (1.89)	2.45 (2.42)	3.05 (3.29)	< 0.01
Insulin resistance (%)^a^	81 (12.8)	128 (18.7)	172 (30.3)	243 (39.8)	< 0.01
Poverty income ratio <1 (%)^a^	145 (25.5)	139 (22.0)	106 (20.2)	92 (16.3)	< 0.01
Serum uric acid (μmol/L)^b^	279.6 (35.7)	339.0 (17.9)	380.7 (23.8)	446.1 (59.5)	< 0.01
Glycohemoglobin (%)^b^	5.4 (0.5)	5.4 (0.4)	5.4 (0.4)	5.5 (0.6)	< 0.01
Hypertension (%)^a^	149 (23.5)	149 (21.8)	154 (27.2)	221 (36.2)	< 0.01
Had at least 12 alcohol drinks/year (%)^a^	455 (78.6)	566 (87.5)	444 (86.2)	483 (85.3)	< 0.01
Smoked at least 100 cigarettes in life (%)^a^	342 (53.9)	342 (49.9)	296 (52.2)	317 (51.9)	0.559

*NHANES 2011–2016 (Men = 2,498).*

*Data are number of subjects (percentage) or medians (interquartile ranges).*

*^a^Chi-square test was used to compare the percentage among participants in different groups.*

*^b^Kruskal–Wallis test was used to compare the median values among participants in different groups.*

*Serum uric acid quartiles: Q1 (uric acid ≤ 309.30 μmol/L), Q2 (309.30 < uric acid ≤ 356.90 μmol/L), Q3 (356.90 < uric acid ≤ 404.50 μmol/L), and Q4 (uric acid > 404.50 μmol/L).*

**TABLE 2:** Clinical characteristics of the study population disaggregated by quartiles of serum uric acid level.

Serum uric acid quartile	Q1	Q2	Q3	Q4	*P*-value
Number of subjects	663	694	648	645	
Age (year)^b^	41 (23)	42 (25)	44 (26)	52 (24)	0.058
Race (%)^a^					< 0.01
Mexican American	108 (16.3)	90 (13.0)	77 (11.9)	69 (10.7)	
Other Hispanic	89 (13.4)	84 (12.1)	67 (10.3)	52 (8.1)	
Non-Hispanic White	231 (34.8)	263 (37.9)	238 (36.7)	294 (45.6)	
Non-Hispanic Black	139 (21.0)	131 (18.9)	140 (21.6)	140 (21.7)	
Other race	96 (14.5)	126 (18.2)	126 (19.4)	90 (14.0)	
Education level (%)^a^					< 0.01
Less than 9th grade	56 (8.4)	63 (9.1)	57 (8.8)	43 (6.7)	
9–11th grade	83 (12.5)	66 (9.5)	73 (11.3)	75 (11.6)	
High school graduate	116 (17.5)	121 (17.4)	119 (18.4)	146 (22.6)	
College degree	203 (30.6)	209 (30.1)	216 (33.3)	225 (34.9)	
College graduate or above	205 (30.9)	235 (33.9)	183 (28.2)	156 (24.2)	
Waist circumference (cm)^b^	86.2 (16.2)	91.0 (19.3)	96.3 (22.0)	102.8 (22.3)	< 0.01
Body mass index (kg/m^2^)^b^	24.9 (6.6)	26.9 (8.6)	28.6 (9.6)	31.3 (11.0)	< 0.01
Cholesterol (mmol/L)^b^	4.71 (1.26)	4.81 (1.24)	4.94 (1.27)	5.12 (1.39)	0.085
Triglyceride (mmol/L)^b^	0.84 (0.57)	0.93 (0.67)	1.03 (0.72)	1.30 (0.96)	< 0.01
Creatinine (μmol/L)^b^	60.1 (15.9)	61.9 (15.0)	64.5 (18.4)	68.1 (21.2)	< 0.01
eGFR (mL/min per 1⋅73m^2^)^b^	100.18 (32.10)	94.81 (27.45)	91.96 (30.86)	83.53 (35.13)	< 0.01
Insulin (mU/L)^b^	7.06 (5.84)	8.16 (7.00)	9.96 (8.66)	11.66 (11.00)	< 0.01
Fasting glucose (mmol/L)^b^	5.22 (0.56)	5.27 (0.67)	5.38 (0.61)	5.50 (0.78)	< 0.01
HOMA-IR^b^	1.64 (1.37)	1.89 (1.84)	2.37 (2.24)	2.84 (3.00)	< 0.01
Insulin resistance (%)^a^	82 (12.4)	131 (18.9)	180 (27.8)	270 (41.9)	< 0.01
Poverty income ratio <1 (%)^a^	166 (27.2)	160 (24.7)	139 (23.3)	149 (25.3)	0.485
Serum uric acid (μmol/L)^b^	208.2 (35.7)	255.8 (23.8)	297.4 (23.8)	362.8 (59.5)	< 0.01
Glycohemoglobin (%)^b^	5.4 (0.5)	5.4 (0.5)	5.4 (0.5)	5.5 (0.5)	0.360
Hypertension (%) ^a^	136 (20.5)	159 (22.9)	199 (30.7)	293 (45.4)	< 0.01
Had at least 12 alcohol drinks/year (%)^a^	359 (62.4)	402 (66.4)	363 (63.4)	344 (59.9)	0.138
Smoked at least 100 cigarettes in life (%)^a^	207 (31.2)	211 (30.4)	204 (31.5)	231 (35.8)	0.149

*NHANES 2011–2016 (Women = 2,650).*

*Data are number of subjects (percentage) or medians (interquartile ranges).*

*^a^Chi-square test was used to compare the percentage among participants in different groups.*

*^b^Kruskal–Wallis test was used to compare the median values among participants in different groups.*

*Serum uric acid quartiles: Q1 (uric acid ≤ 232.00 μmol/L), Q2 (232.00 < uric acid ≤ 273.60 μmol/L), Q3 (273.60 < uric acid ≤ 321.20 μmol/L), and Q4 (uric acid > 321.20 μmol/L).*

**TABLE 3:** Clinical characteristics of the study population in insulin resistance and non-insulin resistance group.

Characteristics	Insulin resistance group	Non-insulin resistance group	*P*-value
Number of subjects	1287	3861	
Age (year)^b^	43 (26)	43 (27)	0.855
Race (%)^a^			< 0.01
Mexican American	231 (17.9)	451 (11.7)	
Other Hispanic	152 (11.8)	415 (10.7)	
Non-Hispanic White	466 (36.2)	1534 (39.7)	
Non-Hispanic Black	279 (21.7)	741 (19.2)	
Other race	159 (12.4)	720 (18.6)	
Education level (%)^a^			< 0.01
Less than 9th grade	119 (9.2)	305 (7.9)	
9–11th grade	188 (14.6)	480 (12.4)	
High school graduate	275 (21.4)	810 (21.0)	
College or AA degree	418 (32.5)	1127 (29.2)	
College graduate or above	287 (22.3)	1139 (29.5)	
Waist circumference (cm)^b^	108 (19.7)	91.7 (18.1)	< 0.01
Body mass index (kg/m^2^)^b^	32.5 (8.8)	25.9 (6.5)	< 0.01
Cholesterol (mmol/L)^b^	4.91 (1.35)	4.86 (1.31)	0.029
Triglyceride (mmol/L)^b^	1.502 (1.14)	0.982 (0.711)	< 0.01
Creatinine (μmol/L)^b^	73.37 (23.87)	73.37 (25.63)	0.578
eGFR (mL/min per 1⋅73m^2^) ^b^	92.42 (31.37)	92.50 (28.65)	0.793
Poverty income ratio <1 (%)^a^	292 (25.1)	804 (22.5)	0.072
Serum uric acid (μmol/L)^b^	345.0 (113.0)	303.3 (101.1)	< 0.01
Glycohemoglobin (%)^b^	5.6 (0.5)	5.4 (0.5)	< 0.01
Hypertension (%)^a^	491 (38.2)	969 (25.1)	< 0.01
Had at least 12 alcohol drinks/year (%)^a^	810 (70.6)	2606 (74.8)	< 0.01
Smoked at least 100 cigarettes in life (%)^a^	537 (41.7)	1613 (41.8)	0.974

*NHANES 2011–2016 (n = 5,148).*

*Data are number of subjects (percentage) or medians (interquartile ranges).*

*^a^Chi-square test was used to compare the percentage of participants with and without insulin resistance.*

*^b^Mann–Whitney U-test was used to compare the median values between participants with and without insulin resistance.*

**FIGURE 2:**
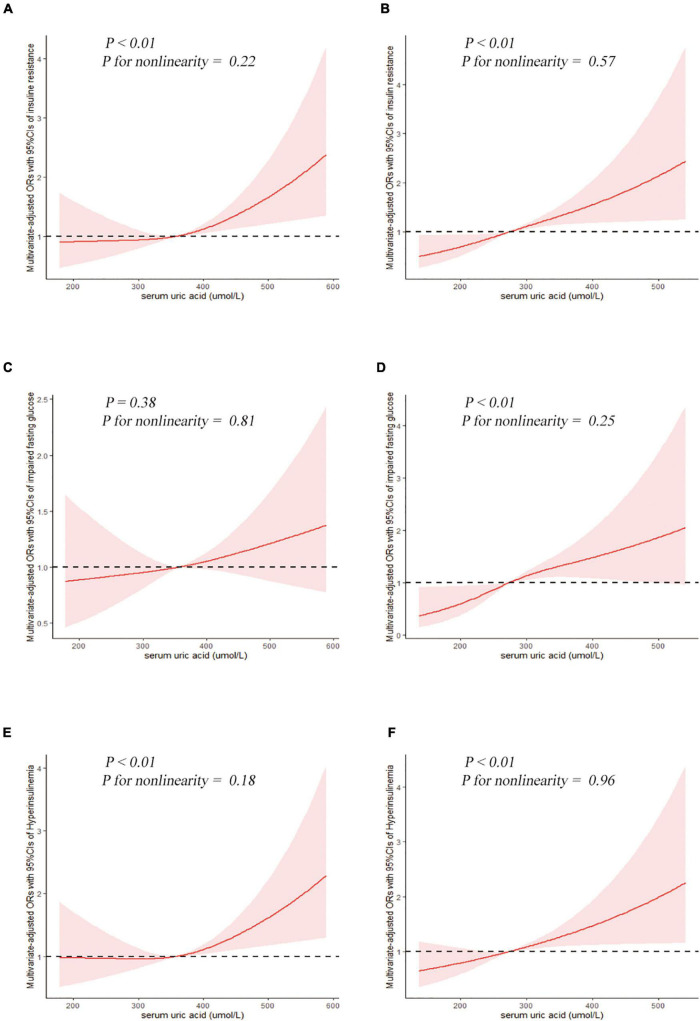
Examination of the dose-response relationship between serum uric acid (μmol/L) and the risk of impaired glucose metabolism by restricted cubic splines model. The restricted cubic splines model adjusted for race, BMI, waist circumference, drinking status, education level, hypertension, total cholesterol, triglyceride, and urate-lowering therapy. Insulin resistance, **(A)** men and **(B)** women. Impaired fasting glucose, **(C)** men and **(D)** women. Hyperinsulinemia, **(E)** men and **(F)** women.

The results of binary logistic regression analysis are presented in Tables 4–8. In men, the crude ORs with 95% confidence intervals (CIs) of IR was 1.6 (1.1–2.3), 3.2 (2.2–4.8), and 4.6 (3.3–6.6) in Q2, Q3, and Q4, respectively, vs. Q1 of SUA. In Model 1, after adjusting for race, the adjusted ORs with 95% CIs were 1.6 (1.1–2.3), 3.2 (2.1–4.8), and 4.8 (3.4–6.7) in Q2, Q3, and Q4, respectively, vs. Q1 of SUA. In Model 2, the multivariate-adjusted ORs with 95% CIs were 1.2 (0.7–1.9), 1.9 (1.1–3.4), and 1.9 (1.1–3.1) in Q2, Q3, and Q4, respectively, vs. Q1 of SUA. In women, the crude ORs with 95% CIs of IR were 1.7 (1.2–2.5), 2.3 (1.4–3.6), and 5.9 (3.8–9.1) in Q2, Q3, and Q4, respectively, vs. Q1 of SUA. In Model 1, the adjusted ORs with 95% CI were 1.9 (1.3–2.7), 2.5 (1.5–3.9), and 6.6 (4.2–10.4) in Q2, Q3, and Q4, respectively, vs. Q1 of SUA. In Model 2, the multivariate-adjusted ORs with 95% CIs were 1.3 (0.7–2.5), 1.3 (0.6–2.5), and 2.2 (1.2–4.3) in Q2, Q3, and Q4, respectively, vs. Q1 of SUA. The dose-response relationships between SUA and IR are presented in Figures 2A,B, **W**hile uric acid levels were positively associated with the risk of IR in both men and women (*P* < 0.01, *P* for non-linearity = 0.22 and 0.57, respectively). The reference points (OR = 1) of SUA in the restricted cubic spline analysis were 357.86 μmol/L for men and 275.01 μmol/L for women, respectively.

**TABLE 4:** Weighted odds ratios (95% confidence intervals) for insulin resistance (HOMA-IR) of participants across quartiles of serum uric acid (Men = 2,498, women = 2,650).

	Case/Participants	Crude^a^	Model 1^a^	Model 2^a^
**Men^†^**				
Q1	635/2,498	1.00 (Ref.)	1.00 (Ref.)	1.00 (Ref.)
Q2	685/2,498	1.6 (1.1–2.3) *	1.6 (1.1–2.3) *	1.2 (0.7–1.9)
Q3	567/2,498	3.2 (2.2–4.8) **	3.2 (2.1–4.8) **	1.9 (1.1–3.4) *
Q4	611/2,498	4.6 (3.3–6.6) **	4.8 (3.4–6.7) **	1.9 (1.1–3.1) *
**Women^†^**				
Q1	663/2,650	1.00 (Ref.)	1.00 (Ref.)	1.00 (Ref.)
Q2	694/2,650	1.7 (1.2–2.5) **	1.9 (1.3–2.7) **	1.3 (0.7–2.5)
Q3	648/2,650	2.3 (1.4–3.6) **	2.5 (1.5–3.9) **	1.3 (0.6–2.5)
Q4	645/2,650	5.9 (3.8–9.1) **	6.6 (4.2–10.4) **	2.2 (1.2–4.3) *

*^a^Calculated using binary logistic regression.*

*^†^Men: Q1 (uric acid ≤ 309.30 μmol/L), Q2 (309.30 < uric acid ≤ 356.90 μmol/L), Q3 (356.90 < uric acid ≤ 404.50 μmol/L), and Q4 (uric acid > 404.50 μmol/L). Women: Q1 (uric acid ≤ 232.00 μmol/L), Q2 (232.00 < uric acid ≤ 273.60 μmol/L), Q3 (273.60 < uric acid ≤ 321.20 μmol/L), and Q4 (uric acid > 321.20 μmol/L).*

*Model 1 adjusted for race.*

*Model 2 adjusted for race, body mass index, waist circumference, drinking status, education level, hypertension, serum triglyceride, total cholesterol, and urate-lowering therapy.*

*P < 0.05.

**P < 0.01.

**TABLE 5:** Weighted odds ratios (95% confidence intervals) for impaired fasting glucose of participants across quartiles of serum uric acid (Men = 2,498, women = 2,650).

	Case/Participants	Crude^a^	Model 1^a^	Model 2^a^
**Men^†^**				
Q1	635/2,498	1.00 (Ref.)	1.00 (Ref.)	1.00 (Ref.)
Q2	685/2,498	0.8 (0.6–1.29)	0.8 (0.5–1.3)	0.8 (0.5–1.4)
Q3	567/2,498	1.7 (1.1–2.56) *	1.7 (1.1–2.6) *	1.2 (0.7–2.1)
Q4	611/2,498	2.1 (1.4–3.03) **	2.1 (1.4–3.1) **	1.4 (0.8–2.2)
**Women^†^**				
Q1	663/2,650	1.00 (Ref.)	1.00 (Ref.)	1.00 (Ref.)
Q2	694/2,650	1.5 (0.8–2.9)	1.5 (0.8–2.9)	1.3 (0.6–3.1)
Q3	648/2,650	1.8 (1.0–3.3)	1.8 (1.0–3.2)	1.5 (0.7–3.1)
Q4	645/2,650	4.9 (2.8–8.7) **	4.8 (2.7–8.5) **	2.7 (1.4–5.5) **

*^a^Calculated using binary logistic regression.*

*^†^Men: Q1 (uric acid ≤ 309.30 μmol/L), Q2 (309.30 < uric acid ≤ 356.90 μmol/L), Q3 (356.90 < uric acid ≤ 404.50 μmol/L), and Q4 (uric acid > 404.50 μmol/L). Women: Q1 (uric acid ≤ 232.00 μmol/L), Q2 (232.00 < uric acid ≤ 273.60 μmol/L), Q3 (273.60 < uric acid ≤ 321.20 μmol/L), and Q4 (uric acid > 321.20 μmol/L).*

*Model 1 adjusted for race.*

*Model 2 adjusted for race, body mass index, waist circumference, drinking status, education level, hypertension, serum triglyceride, total cholesterol, and urate-lowering therapy.*

*P < 0.05.

**P < 0.01.

**TABLE 6:** Weighted odds ratios (95% confidence intervals) for hyperinsulinemia of participants across quartiles of serum uric acid (Men = 2,498, women = 2,650).

	Case/Participants	Crude^a^	Model 1^a^	Model 2^a^
**Men^†^**				
Q1	635/2,498	1.00 (Ref.)	1.00 (Ref.)	1.00 (Ref.)
Q2	685/2,498	1.5 (1.0–2.2) *	1.5 (1.0–2.2) *	1.2 (0.7–1.9)
Q3	567/2,498	2.8 (1.8–4.3) **	2.8 (1.8–4.3) **	1.6 (0.9–2.9)
Q4	611/2,498	4.4 (3.1–6.3) **	4.5 (3.1–6.5) **	1.8 (1.1–2.8) *
**Women^†^**				
Q1	663/2,650	1.00 (Ref.)	1.00 (Ref.)	1.00 (Ref.)
Q2	694/2,650	1.7 (1.2–2.4) **	1.8 (1.3–2.6) **	1.3 (0.7–2.4)
Q3	648/2,650	2.3 (1.5–3.5) **	2.4 (1.5–3.8) **	1.3 (0.7–2.4)
Q4	645/2,650	5.4 (3.6–8.0) **	5.9 (4.0–8.9) **	2.0 (1.1–3.8) *

*^a^Calculated using binary logistic regression.*

*^†^Men: Q1 (uric acid ≤ 309.30 μmol/L), Q2 (309.30 < uric acid ≤ 356.90 μmol/L), Q3 (356.90 < uric acid ≤ 404.50 μmol/L), and Q4 (uric acid > 404.50 μmol/L). Women: Q1 (uric acid ≤ 232.00 μmol/L), Q2 (232.00 < uric acid ≤ 273.60 μmol/L), Q3 (273.60 < uric acid ≤ 321.20 μmol/L), and Q4 (uric acid > 321.20 μmol/L).*

*Model 1 adjusted for race.*

*Model 2 adjusted for race, body mass index, waist circumference, drinking status, education level, hypertension, serum triglyceride, total cholesterol, and urate-lowering therapy.*

*P < 0.05.

**P < 0.01.

**TABLE 7:** Weighted odds ratios (95% confidence intervals) for insulin resistance, impaired fasting glucose, and hyperinsulinemia of participants with the increase of per standard deviation uric acid (Men = 2,498, women = 2,650).

	Model 2^a^
**Men**	
Insulin resistance	1.2 (1.0–1.5) *
Impaired fasting glucose	1.1 (0.9–1.3)
Hyperinsulinemia	1.2 (1.0–1.4) *
**Women**	
Insulin resistance	1.3 (1.1–1.7) **
Impaired fasting glucose	1.3 (1.1–1.6) **
Hyperinsulinemia	1.3 (1.0–1.5) *

*^a^Calculated using regression analysis.*

*Model 2 adjusted for race, body mass index, waist circumference, drinking status, education level, hypertension, serum triglyceride, total cholesterol, and urate-lowering therapy.*

*P < 0.05.

**P < 0.01.

**TABLE 8:** Weighted odds ratios (95% confidence intervals) for insulin resistance (HOMA-IR) of participants across quartiles of serum uric acid, stratified analysis by menopause (Pre-menopausal women = 1,245; Post-menopausal women = 1,122).

	Case/Participants	Crude^a^	Model 1^a^	Model 2^a^
**Pre-menopausal women^†^**				
Q1	322/1,245	1.00 (Ref.)	1.00 (Ref.)	1.00 (Ref.)
Q2	354/1,245	2.2 (1.2–4.0) *	2.5 (1.4–4.7) **	2.0 (1.1–3.7) *
Q3	258/1,245	3.2 (1.8–5.7) **	3.8 (2.1–6.7) **	2.2 (1.1–4.4) *
Q4	311/1,245	8.2 (5.2–13.0) **	9.5 (6.1–14.9) **	3.5 (2.0–6.0) **
*P* for trend		< 0.01	< 0.01	< 0.01
**Post-menopausal women^†^**				
Q1	303/1,122	1.00 (Ref.)	1.00 (Ref.)	1.00 (Ref.)
Q2	285/1,122	1.1 (0.5–2.2)	1.1 (0.5–2.2)	0.6 (0.3–1.3)
Q3	257/1,122	1.7 (0.8–3.6)	1.7 (0.8–3.7)	0.9 (0.4–2.0)
Q4	277/1,122	4.1 (2.0–8.1) **	4.2 (2.1–8.6) **	1.9 (0.8–4.4)
*P* for trend		< 0.01	< 0.01	0.082

*^a^Calculated using binary logistic regression.*

*^†^Pre-menopausal women: Q1 (uric acid ≤ 226.00 μmol/L), Q2 (226.00 < uric acid ≤ 267.70 μmol/L), Q3 (267.70 < uric acid ≤ 303.30 μmol/L), and Q4 (uric acid > 303.30 μmol/L).*

*Post-menopausal women: Q1 (uric acid ≤ 249.80 μmol/L), Q2 (249.80 < uric acid ≤ 291.50 μmol/L), Q3 (291.50 < uric acid ≤ 339.00 μmol/L), and Q4 (uric acid > 339.00 μmol/L).*

*Model 1 adjusted for race.*

*Model 2 adjusted for race, body mass index, waist circumference, drinking status, education level, hypertension, serum triglyceride, total cholesterol, and urate-lowering therapy.*

*P < 0.05.

**P < 0.01.

The results of binary logistic regression analysis between SUA levels and the risk of impaired fasting glucose are shown in Table 5. In the full-adjusted Model 2, the ORs with 95% CIs were 0.8 (0.5–1.4), 1.2 (0.7–2.1), and 1.4 (0.8–2.2) in Q2, Q3, and Q4, respectively, vs. Q1 of SUA in men, and the ORs with 95%CIs were 1.3 (0.6–3.1), 1.5 (0.7–3.1), and 2.7 (1.4–5.5) in Q2, Q3, and Q4, respectively, vs. Q1 of SUA in women. The results of the restricted cubic spline analysis between SUA and the risk of impaired fasting glucose are shown in Figures 2C,D. SUA levels were positively associated with the risk of IFG in women (*P* < 0.01) but not in men (*P* = 0.38).

The results of binary logistic regression analysis between SUA levels and the risk of hyperinsulinemia are presented in Table 6. In men, the ORs with 95% CIs were 1.5 (1.0–2.2), 2.8 (1.8–4.3), and 4.5 (3.1–6.5) in Q2, Q3, and Q4, respectively, vs. Q1 of SUA in Model 1, while in Model 2, the ORs with 95% CIs were 1.2 (0.7–1.9), 1.6 (0.9–2.9), and 1.8 (1.1–2.8) in Q2, Q3, and Q4, respectively, vs. Q1 of SUA. In women, the ORs with 95% CIs were 1.8 (1.3–2.6), 2.4 (1.5–3.8), and 5.9 (4.0–8.9) in Q2, Q3, and Q4, respectively, vs. Q1 of SUA in Model 1, while in the full-adjusted Model 2, the ORs with 95% CIs were 1.3 (0.7–2.4), 1.3 (0.7–2.4), and 2.0 (1.1–3.8) in Q2, Q3, and Q4, respectively, vs. Q1 of SUA. The results of the restricted cubic spline analysis between SUA and the risk of hyperinsulinemia are presented in Figures 2E,F. Uric acid levels were positively associated with the risk of hyperinsulinemia in both men and women (*P* < 0.01, *P* for non-linearity = 0.18 and 0.96, respectively).

The OR values with per SD increase in SUA are presented in Table 7. In men, SUA was positively associated with the risk of IR and hyperinsulinemia; the ORs with per SD increase in SUA was 1.2 (1.0–1.5) and 1.2 (1.0–1.4), respectively. In women, SUA was positively associated with the risk of IR, IFG, and hyperinsulinemia; the ORs with per SD increase in SUA was 1.3 (1.1–1.7), 1.3 (1.1–1.6), and 1.3 (1.0–1.5), respectively.

The results of stratified analysis by menopausal status are presented in Table 8. In pre-menopausal women aged ≥20 years, the ORs with 95% CIs were 2.0 (1.1–3.7), 2.2 (1.1–4.4), and 3.5 (2.0–6.0) in Q2, Q3, and Q4, respectively, vs. Q1 of SUA in Model 2, while the ORs among different uric acid groups gradually increased (*P* for trend < 0.01). In the post-menopausal group, the results were significant in the crude model and Model 1 but became insignificant after full adjustment for the confounding factors.

Further sensitivity analysis was performed by excluding participants diagnosed with metabolic syndrome, finally including 4,270 participants. After full adjustment for the same confounding factors, the relationship between SUA and IR continued to be significant (Table 9). Compared with the lowest quartile of SUA, the OR (95% CIs) of insulin resistance in the highest quartile was 1.6 (1.1–2.3) and 1.9 (1.1–3.5) in men and women, respectively.

**TABLE 9:** Weighted odds ratios (95% confidence intervals) for insulin resistance (HOMA-IR) of participants without metabolic syndrome across quartiles of serum uric acid (Men = 2,124, Women = 2,146).

		Crude^a^	Model 1^a^	Model 2^a^
**Males^†^**				
Q1	584/2,124	1.00 (Ref.)	1.00 (Ref.)	1.00 (Ref.)
Q2	538/2,124	1.4 (0.9–2.3) *	1.4 (0.9–2.3)	1.2 (0.7–1.9)
Q3	493/2,124	2.1 (1.4–3.1) **	2.1 (1.4–3.2) **	1.3 (0.8–2.1)
Q4	509/2,124	3.4 (2.3–4.9) **	3.4 (2.4–5.0) **	1.6 (1.1–2.3) *
**Females^†^**				
Q1	607/2,146	1.00 (Ref.)	1.00 (Ref.)	1.00 (Ref.)
Q2	515/2,146	1.3 (0.9–1.9)	1.4 (1.0–2.1)	1.3 (0.7–2.2)
Q3	503/2,146	2.1 (1.4–3.1) **	2.2 (1.4–3.4) **	1.7 (1.0–2.9)
Q4	521/2,146	3.4 (2.1–5.6) **	3.7 (2.2–6.1) **	1.9 (1.1–3.5) *

*^a^Calculated using binary logistic regression.*

*^†^Males: Q1 (uric acid ≤ 309.30 μmol/L), Q2 (309.30 < uric acid ≤ 350.90 μmol/L), Q3 (350.90 < uric acid ≤ 398.50 μmol/L), and Q4 (uric acid > 398.50 μmol/L). Females: Q1 (uric acid ≤ 232.00 μmol/L), Q2 (232.00 < uric acid ≤ 267.70 μmol/L), Q3 (267.70 < uric acid ≤ 309.30 μmol/L), and Q4 (uric acid > 309.30 μmol/L).*

*Model 1 adjusted for race.*

*Model 2 adjusted for race, body mass index, waist circumference, drinking status, education level, hypertension, serum triglyceride, total cholesterol, and urate-lowering therapy.*

*P < 0.05.

**P < 0.01.

The results of linear regression analysis between SUA levels and HOMA-IR are presented in Supplementary Table 1. In men, the coefficients (β) with 95% CIs were 0.09 (−0.15 to 0.33), 0.37 (0.00–0.75), and 0.68 (0.26–1.09) in Q2, Q3, and Q4, respectively, vs. Q1 of SUA in Model 2. In women, the coefficients (β) with 95% CIs were 0.07 (−0.14 to 0.28), 0.15 (−0.15 to 0.45), and 0.41 (0.11–0.72) in Q2, Q3, and Q4, respectively, vs. Q1 of SUA in Model 2.

The results of stratified analysis based on BMI, waist circumference, drinking status, educational levels, hypertension, serum triglyceride, and total cholesterol are summarized in Supplementary Tables 2–8. We found that the association between SUA and IR was more pronounced among participants with obesity, bigger waist circumference, hyperlipidemia, and those with a college degree.

## Discussion

In this study, we combined data from the NHANES 2011–2016 to investigate the relationship between SUA and impaired glucose metabolism in people without diabetes. To the best of our knowledge, this is the first study that used restricted cubic spline analysis and stratified analysis by menopausal status to investigate the above association with a large-scale sample. We found that SUA was positively correlated with IR risk in all individuals, except for post-menopausal individuals. In women, elevated UA levels were positively correlated with increased risk of IFG and hyperinsulinemia, while in men, elevated UA levels were positively correlated with increased risk of hyperinsulinemia, but not IFG. Restricted cubic spline revealed that the concentrations of SUA were positively associated with the risk of IR and hyperinsulinemia in both men and women, and the above association seemed more pronounced among women than men.

The pathophysiological mechanism for the development of IR remains unclear; however, both genetic factors and environmental factors are involved. Previous experimental studies have suggested that increasing adipose tissue can result in IR through chronic low-grade inflammation and an imbalance of pro-inflammatory and anti-inflammatory adipokines (13). While the mechanism of the relationship between SUA and IR has not yet been fully elucidated, several possible explanations have been proposed. Primarily, elevated SUA directly induces IR by increasing the production of reactive oxygen species (ROS) and inhibiting insulin receptor substrate 1 (IRS1) and protein kinase B (Akt) insulin signaling. Antioxidant N-acetyl-L-cysteine could inhibit ROS production and block hyperuricemia-induced IRS1 activation and Akt inhibition (30). In addition, uric acid could induce IR by regulating activation of the NLRP3 inflammasome. Urate-lowering therapy with allopurinol suppresses the expression of the NLRP3 inflammasome, and the knockdown of NLRP3 expression improves insulin signaling by reducing IRS1 phosphorylation and enhancing Akt phosphorylation (31). Reduction of endothelial nitric oxide supply and bioavailability, and activation of NADPH oxidase, also medicates the IR (32). On the other hand, IR is inversely correlated with the renal clearance of SUA by enhancing renal tubular reabsorption of uric acid (11, 33). Hyperinsulinemia could increase the activation of the hexose phosphate, which promotes the biosynthesis of purine and increases the rate of uricogenesis, eventually resulting in hyperuricemia (19).

In their study, Choi et al. found that SUA levels increased linearly with HOMA-IR levels in the general United States adult population (34). A study conducted in 2020 revealed that elevated SUA was associated with a higher risk of IR in type 2 diabetes and the above association was more pronounced in female patients (18). A study conducted in 2011 found that SUA was positively correlated with IFG only in women, which was consistent with our results. The lower visceral glucocorticoid receptor density in women leads to a reduced visceral fat mass compared to that in men, and the differences in fat distribution between men and women partly explain the gender differences (35). However, in Ma’s study, which only involved patients with newly diagnosed type 2 diabetes, there was no significant association between uric acid and HbA1c in the low insulin group, while an inverse relationship was found in the high insulin group (19). A study conducted by Balikcioglu et al. involving 82 obese adolescents found that uric acid was positively correlated with HOMA-IR in men but not in women (36). In another study, which involved healthy people without diabetes (*n* = 102), prediabetes (*n* = 98), and diabetes (*n* = 110), an inverse association was found between the SUA and fasting glucose levels and the prevalence of diabetes (37). These observations might be explained by higher renal clearance of uric acid in adult diabetic patients (38). The following reasons could potentially explain the differences in the above studies: (1) the participants involved in previous studies had different characteristics. For instance, only newly diagnosed type 2 diabetes patients were involved in Ma’s study (19) and only obese adolescents in Balikcioglu’s study (36). Besides, the healthy individuals and diabetic patients in Ali’s study (37) came from the same university or hospital in Bangladesh, while in the present study, we only included individuals without diabetes. (2) The research methods, statistical analysis, and adjusted confounding factors differed in the above-mentioned research. Only age, BMI, blood pressure, triglyceride, and creatinine were adjusted in Ma’s study (19), while branched-chain amino acid (BCAA) levels and products of BCAA catabolism were additionally adjusted in Balikcioglu’s study (36). Besides, age, gender, serum lipid profile, albumin, and total protein were adjusted in Ali’s study (37), while in the present study, we only additionally adjusted for race, waist circumference, drinking status, education level, and urate-lowering therapy. Analysis was also conducted separately by gender. To sum up, different participants, research methods, and the adjusted confounding factors might explain the inconsistent results.

Previous studies have reported the influence of menopausal transition on the changes in SUA. Cho et al. found that the prevalence of hyperuricemia significantly increases as the menopausal stage increases (39). Ahmed et al. suggested that a reduction of estrogen levels in post-menopausal women leads to a higher risk of type 2 diabetes mellitus (T2DM) (40). Another study found that menopause is often related to the accumulation of visceral adipose tissue (VAT), which may provoke IR and result in hyperinsulinemia (41). Also, menopausal hormone therapy may improve insulin secretion and sensitivity, thus decreasing the risk of T2DM (42). Due to the influence of menopause, we further performed a stratified analysis based on menopausal status to explore the above relationship in women without diabetes.

Our results showed that SUA was significantly associated with IR only in pre-menopausal women aged ≥ 20 years but not in the post-menopausal group. No previous study found this phenomenon. However, the reason for this result remained poorly understood. The sex hormone alteration in the post-menopausal period, decreasing serum estrogen and sex hormone-binding globulin, and elevated free testosterone levels could all lead to the accumulation of VAT (43, 44). As the accumulation of VAT is the main predictor of IR, we speculate that the production of pro-inflammatory factors by adipocytes and chronic low-grade inflammation state might weaken the effect of uric acid on IR in post-menopausal women. Future studies are needed to confirm the differences in the relationship between SUA and IR in women with different menopausal statuses and explore the causes of the above phenomenon.

We used a large national representative sample among the general United States population, which increased the statistical strength and confirmed the reliability of the reported results. We fully adjusted the analyses for the potential confounding factors and analyzed the association with different statistical methods. To the best of our knowledge, this is the first study that used binary logistic regression analysis to investigate the relationship between SUA and glucose metabolism, and restricted cubic splines and stratified analysis by menopausal status to explore the relationship between SUA and glucose metabolism with a large-scale nationwide sample. Due to the differences in uric acid levels between men and women, the analyses were conducted separately by gender. Therefore, it is helpful to explore the gender difference in the relationship between uric acid and impaired glucose metabolism. In the present study, we only included people without diabetes to avoid the effect of oral hypoglycemic drugs and exogenous insulin. To minimize the influence of metabolic syndrome, we performed a sensitivity analysis by excluding participants without metabolic syndrome, and the obtained results were still stable.

The present study has the following limitations: primarily, as this was a cross-sectional study, it was difficult to determine causality, so further experimental studies and large-scale prospective research are required to confirm the causality. Furthermore, the serum variables in our study were only measured once, while uric acid, fasting glucose, and insulin were not static but dynamic variables, which may be affected by the diet and cause some bias. In addition, due to the limited data, it was impossible to include all potential confounding factors. We also did not analyze the relationship between SUA and other indexes reflecting the function of islet β cells, such as the C peptide. Meanwhile, we used data from NHANES 2011 to 2016, some of which may be considered outdated, considering the changes that may have occurred due to changes in lifestyle and food habits.

With improved living conditions, the prevalence of hyperuricemia is gradually increasing. It is generally agreed that gout patients should initiate urate-lowering therapy; however, for asymptomatic hyperuricemia patients, most countries do not recommend initiating urate-lowering therapy due to the potentially serious adverse effects. Our results suggest that more attention should be paid to the SUA level; for people with unexplained impaired glucose metabolism, screening for SUA might be necessary. For people with elevated SUA levels, even those without urate-lowering therapy, professional advice on how to control uric acid levels, such as weight loss, avoidance of alcohol, sugar-sweetened drinks, and seafood, might be helpful.

## Conclusion

Our study suggests that SUA levels might be positively associated with impaired glucose metabolism in the general United States adult population without diabetes. We found that uric acid increased the risk of IR in pre-menopausal women aged ≥20 years rather than in post-menopausal women. We hope that these results can provide valuable information for the screening and treatment of impaired glucose metabolism and screening of SUA. Future studies are needed to confirm the differences in the relationship between SUA and IR in women with different menopausal statuses and explore the causes of the above phenomenon.

## Data Availability Statement

The original contributions presented in the study are included in the article/Supplementary Material, further inquiries can be directed to the corresponding authors.

## Ethics Statement

The studies involving human participants were reviewed and approved by the National Center for Health Statistics’ Research Ethics Review Board for all data collection protocols. The patients/participants provided their written informed consent to participate in this study. Written informed consent was obtained from the individual(s) for the publication of any potentially identifiable images or data included in this article.

## Author Contributions

YH: conceptualization, resources, and writing−original draft. YC and HD: data curation. YH, XH, and HD: formal analysis. YZ and XZ: funding acquisition and writing−review and editing. YH and YY: investigation. YH and JW: methodology. YC and YH: software. YZ: supervision and validation. YH, JW, and HD: visualization. All authors helped to perform the research and read and approved the final manuscript.

## Conflict of Interest

The authors declare that the research was conducted in the absence of any commercial or financial relationships that could be construed as a potential conflict of interest.

## Publisher’s Note

All claims expressed in this article are solely those of the authors and do not necessarily represent those of their affiliated organizations, or those of the publisher, the editors and the reviewers. Any product that may be evaluated in this article, or claim that may be made by its manufacturer, is not guaranteed or endorsed by the publisher.

## Abbreviations

NHANES: National Health and Nutrition Examination Surveys
SUA: serum uric acid
BMI: body mass index
ORs: odds ratios
CIs: confidence intervals
eGFR: estimation of the glomerular filtration rate
ROS: reactive oxygen species
PIR: poverty income ratio
IFG: Impaired fasting glucose
HOMA-IR: homeostasis model assessment of insulin resistance
IRS1: insulin receptor substrate 1
Akt: protein kinase B.
