# Supplementary material for: Dose-Response Relationship of Uric Acid With Fasting Glucose, Insulin, and Insulin Resistance in a United States Cohort of 5,148 Non-diabetic People
Source: Front Med (Lausanne). 2022 Jun 9;9:905085. doi: 10.3389/fmed.2022.905085 (PMC9218264; doi:10.3389/fmed.2022.905085)
Supplement: Supplementary file 1 [file Data_Sheet_1.docx]

**Table S1**. Weighted Regression coefficients (β) and 95% confidence intervals of association between quartiles of serum uric acid and HOMA-IR (Men = 2498, women = 2650).

|  | Case/Participants | Crude^1^ | Model 1^1^ | Model 2^1^ |
| --- | --- | --- | --- | --- |
| Men† |  |  |  |  |
| Q1 | 635/2498 | 1.00 (Ref.) | 1.00 (Ref.) | 1.00 (Ref.) |
| Q2 | 685/2498 | 0.34 (0.03-0.65) * | 0.34 (0.03-0.65) * | 0.09 (-0.15-0.33) |
| Q3 | 567/2498 | 1.14 (0.77-1.50) ** | 1.12 (0.75-1.49) ** | 0.37 (0.00-0.75) |
| Q4 | 611/2498 | 1.95 (1.48-2.41) ** | 1.97 (1.50-2.43) ** | 0.68 (0.26-1.09) ** |
| Women† |  |  |  |  |
| Q1 | 663/2650 | 1.00 (Ref.) | 1.00 (Ref.) | 1.00 (Ref.) |
| Q2 | 694/2650 | 0.33 (0.12-0.54) ** | 0.39 (0.17-0.60) ** | 0.07 (-0.14-0.28) |
| Q3 | 648/2650 | 0.71 (0.43-0.97) ** | 0.76 (0.48-1.03) ** | 0.15 (-0.15-0.45) |
| Q4 | 645/2650 | 1.70 (1.30-2.09) ** | 1.76 (1.37-2.15) ** | 0.41 (0.11-0.72) ** |
| 1 Calculated using linear regression analysis.  † Men: Q1 (uric acid ≤ 309.30μmol/L), Q2 (309.30 < uric acid ≤ 356.90μmol/L), Q3 (356.90< uric acid ≤ 404.50μmol/L), Q4 (uric acid > 404.50μmol/L).  Women: Q1 (uric acid ≤ 232.00μmol/L), Q2 (232.00 < uric acid ≤ 273.60μmol/L), Q3 (273.60< uric acid ≤ 321.20μmol/L), Q4 (uric acid > 321.20μmol/L).  Model 1 adjusted for race.  Model 2 adjusted for race, body mass index, waist circumference, drinking status, education level, hypertension, serum triglyceride, total cholesterol, urate lowering therapy.  * *P* < 0.05  ** *P* < 0.01 | | | | |

**Table S2**. Weighted odds ratios (95% confidence intervals) for insulin resistance (HOMA-IR) of participants across quartiles of serum uric acid, stratified analysis by BMI. (BMI < 25 kg/m^2^; n = 1731; 25 ≤BMI ≤ 30 kg/m^2^, n = 1675; BMI > 30 kg/m^2^; n = 1701.).

|  | Crude^1^ | Model 1^1^ | Model 2^1^ |
| --- | --- | --- | --- |
| BMI < 25kg/m^2^ |  |  |  |
| Q1 | 1.00 (Ref.) | 1.00 (Ref.) | 1.00 (Ref.) |
| Q2 | 0.5 (0.2-1.0) * | 0.5 (0.2-1.0) * | 0.5 (0.2-1.2) |
| Q3 | 0.8 (0.5-1.5) | 0.8 (0.4-1.5) | 0.8 (0.4-1.7) |
| Q4 | 0.6 (0.2-1.4) | 0.6 (0.3-1.4) | 0.4 (0.1-1.0) |
| 25 ≤BMI ≤ 30 kg/m^2^ |  |  |  |
| Q1 | 1.00 (Ref.) | 1.00 (Ref.) | 1.00 (Ref.) |
| Q2 | 1.1 (0.7-1.8) | 1.2 (0.7-1.9) | 1.1 (0.6-1.9) |
| Q3 | 2.0 (1.3-3.1) ** | 2.0 (1.3-3.1) ** | 1.7 (1.0-2.8) |
| Q4 | 2.3 (1.5-3.7) ** | 2.4 (1.5-3.7) ** | 1.8 (1.1-3.0) * |
| BMI > 30 kg/m^2^ |  |  |  |
| Q1 | 1.00 (Ref.) | 1.00 (Ref.) | 1.00 (Ref.) |
| Q2 | 2.3 (1.5-3.7) ** | 2.4 (1.5-3.8) ** | 2.5 (1.4-4.6) ** |
| Q3 | 2.6 (1.7-4.0) ** | 2.7 (1.8-4.1) ** | 2.7 (1.6-4.5) ** |
| Q4 | 5.0 (3.1-7.9) ** | 5.4 (3.4-8.4) ** | 4.4 (2.6-7.5) ** |

1 Calculated using binary logistic regression.

Model 1 adjusted for race.

Model 2 adjusted for race, body mass index, waist circumference, drinking status, education level, hypertension, serum triglyceride, total cholesterol, urate lowering therapy.

* *P* < 0.05

** *P* < 0.01

**Table S3**. Weighted odds ratios (95% confidence intervals) for insulin resistance (HOMA-IR) of participants across quartiles of serum uric acid, stratified analysis by waist circumference (WC). (WC <88cm for women and WC <102cm for men; n = 2469; WC≥88cm for women and WC ≥102cm for men; n = 2491.).

|  | Crude^1^ | Model 1^1^ | Model 2^1^ |
| --- | --- | --- | --- |
| Normal WC |  |  |  |
| Q1 | 1.00 (Ref.) | 1.00 (Ref.) | 1.00 (Ref.) |
| Q2 | 0.8 (0.4-1.5) | 0.8 (0.5-1.5) | 0.8 (0.4-1.5) |
| Q3 | 1.8 (1.0-3.1) * | 1.8 (1.0-3.2) * | 1.3 (0.7-2.4) |
| Q4 | 1.4 (0.9-2.2) | 1.5 (1.0-2.4) | 0.8 (0.5-1.5) |
| Bigger WC |  |  |  |
| Q1 | 1.00 (Ref.) | 1.00 (Ref.) | 1.00 (Ref.) |
| Q2 | 2.0 (1.3-3.1) ** | 2.0 (1.3-3.2) ** | 1.6 (0.9-2.8) |
| Q3 | 2.5 (1.7-3.8) ** | 2.6 (1.7-3.9) ** | 1.9 (1.1-3.1) * |
| Q4 | 5.0 (3.2-7.7) ** | 5.3 (3.4-8.1) ** | 3.0 (1.7-5.0) ** |

1 Calculated using binary logistic regression.

Model 1 adjusted for race.

Model 2 adjusted for race, body mass index, waist circumference, drinking status, education level, hypertension, serum triglyceride, total cholesterol, urate lowering therapy.

* *P* < 0.05

** *P* < 0.01

**Table S4**. Weighted odds ratios (95% confidence intervals) for insulin resistance (HOMA-IR) of participants across quartiles of serum uric acid, stratified analysis by hypertension. (Hypertension; n = 1460; non-hypertension; n = 3688.).

|  | Crude^1^ | Model 1^1^ | Model 2^1^ |
| --- | --- | --- | --- |
| Hypertension |  |  |  |
| Q1 | 1.00 (Ref.) | 1.00 (Ref.) | 1.00 (Ref.) |
| Q2 | 1.9 (1.1-3.6) * | 1.9 (1.0-3.6) * | 1.6 (0.7-3.7) |
| Q3 | 2.8 (1.5-5.2) ** | 2.7 (1.5-5.1) ** | 2.0 (0.8-4.7) |
| Q4 | 3.9 (2.2-6.9) ** | 4.1 (2.3-7.1) ** | 2.5 (1.2-5.2) * |
| Non-hypertension |  |  |  |
| Q1 | 1.00 (Ref.) | 1.00 (Ref.) | 1.00 (Ref.) |
| Q2 | 1.5 (1.1-2.1) ** | 1.6 (1.2-2.1) ** | 1.1 (0.7-1.6) |
| Q3 | 2.6 (1.9-3.5) ** | 2.7 (2.0-3.7) ** | 1.4 (1.0-2.0) |
| Q4 | 5.5 (4.0-7.7) ** | 6.0 (4.3-8.3) ** | 1.8 (1.2-2.7) ** |

1 Calculated using binary logistic regression.

Model 1 adjusted for race.

Model 2 adjusted for race, body mass index, waist circumference, drinking status, education level, serum triglyceride, total cholesterol, urate lowering therapy.

* *P* < 0.05

** *P* < 0.01

**Table S5**. Weighted odds ratios (95% confidence intervals) for insulin resistance (HOMA-IR) of participants across quartiles of serum uric acid, stratified analysis by education levels. (College and above; n = 2971; high school and below; n = 2177.).

|  | Crude^1^ | Model 1^1^ | Model 2^1^ |
| --- | --- | --- | --- |
| College and above |  |  |  |
| Q1 | 1.00 (Ref.) | 1.00 (Ref.) | 1.00 (Ref.) |
| Q2 | 2.0 (1.3-2.9) ** | 2.0 (1.4-3.0) ** | 1.5 (0.8-2.8) |
| Q3 | 3.0 (2.0-4.5) ** | 3.1 (2.1-4.6) ** | 1.6 (0.8-3.2) |
| Q4 | 6.6 (4.3-10.1) ** | 6.9 (4.5-10.6) ** | 2.3 (1.3-4.2) ** |
| High school and below |  |  |  |
| Q1 | 1.00 (Ref.) | 1.00 (Ref.) | 1.00 (Ref.) |
| Q2 | 1.3 (09-1.9) | 1.3 (0.9-2.0) | 1.0 (0.6-1.8) |
| Q3 | 2.4 (1.7-3.3) ** | 2.5 (1.8-3.5) ** | 1.6 (1.0-2.6) * |
| Q4 | 3.8 (2.7-5.4) ** | 4.1 (2.9-5.7) ** | 1.8 (1.1-3.0) * |

1 Calculated using binary logistic regression.

Model 1 adjusted for race.

Model 2 adjusted for race, body mass index, waist circumference, drinking status, education level, hypertension, serum triglyceride, total cholesterol, urate lowering therapy.

* *P* < 0.05

** *P* < 0.01

**Table S6**. Weighted odds ratios (95% confidence intervals) for insulin resistance (HOMA-IR) of participants across quartiles of serum uric acid, stratified analysis by drinking status. (Drinking; n = 3416; non-drinking; n = 1218.).

|  | Crude^1^ | Model 1^1^ | Model 2^1^ |
| --- | --- | --- | --- |
| Drinking |  |  |  |
| Q1 | 1.00 (Ref.) | 1.00 (Ref.) | 1.00 (Ref.) |
| Q2 | 1.8 (1.2-2.7) ** | 1.8 (1.2-2.7) ** | 1.3 (0.8-2.1) |
| Q3 | 2.8 (1.8-4.2) ** | 2.8 (1.8-4.3) ** | 1.5 (0.9-2.5) |
| Q4 | 5.0 (3.3-7.5) ** | 5.2 (3.5-7.8) ** | 1.8 (1.1-3.1) * |
| Non-drinking |  |  |  |
| Q1 | 1.00 (Ref.) | 1.00 (Ref.) | 1.00 (Ref.) |
| Q2 | 1.3 (0.8-2.4) | 1.5 (0.8-2.6) | 1.0 (0.5-1.9) |
| Q3 | 3.4 (2.2-5.4) ** | 3.7 (2.3-5.8) ** | 1.9 (1.1-3.2) * |
| Q4 | 6.9 (4.3-11.1) ** | 7.6 (4.7-12.5) ** | 3.2 (1.7-5.9) ** |

1 Calculated using binary logistic regression.

Model 1 adjusted for race.

Model 2 adjusted for race, body mass index, waist circumference, education level, hypertension, serum triglyceride, total cholesterol, urate lowering therapy.

* *P* < 0.05

** *P* < 0.01

**Table S7**. Weighted odds ratios (95% confidence intervals) for insulin resistance (HOMA-IR) of participants across quartiles of serum uric acid, stratified analysis by triglyceride (TG). (TG≥1.7mmol/L; n = 1137; TG<1.7mmol/L; n = 4006.).

|  | Crude^1^ | Model 1^1^ | Model 2^1^ |
| --- | --- | --- | --- |
| TG≥1.7mmol/L |  |  |  |
| Q1 | 1.00 (Ref.) | 1.00 (Ref.) | 1.00 (Ref.) |
| Q2 | 2.2 (1.2-4.0) ** | 2.3 (1.2-4.2) * | 1.6 (0.7-4.0) |
| Q3 | 2.7 (1.4-5.5) ** | 2.8 (1.4-5.7) ** | 2.1 (0.8-5.8) |
| Q4 | 5.5 (3.0-9.8) ** | 5.9 (3.2-11.0) ** | 3.6 (1.5-8.9) ** |
| TG<1.7mmol/L |  |  |  |
| Q1 | 1.00 (Ref.) | 1.00 (Ref.) | 1.00 (Ref.) |
| Q2 | 1.4 (1.0-1.9) | 1.4 (1.0-2.0) * | 1.1 (0.7-1.9) |
| Q3 | 2.4 (1.7-3.5) ** | 2.5 (1.7-3.6) ** | 1.4 (0.9-2.3) |
| Q4 | 3.9 (2.6-5.8) ** | 4.1 (2.8-6.2) ** | 1.5 (0.9-2.4) |

1 Calculated using binary logistic regression.

Model 1 adjusted for race.

Model 2 adjusted for race, body mass index, waist circumference, drinking status, education level, hypertension, serum triglyceride, total cholesterol, urate lowering therapy.

* *P* < 0.05

** *P* < 0.01

**Table S8**. Weighted odds ratios (95% confidence intervals) for insulin resistance (HOMA-IR) of participants across quartiles of serum uric acid, stratified analysis by total cholesterol (TC). (TC≥ 5.2mmol/L; n = 2027; TC< 5.2 mmol/L; n = 3121.).

|  | Crude^1^ | Model 1^1^ | Model 2^1^ |
| --- | --- | --- | --- |
| TC≥ 5.2mmol/L |  |  |  |
| Q1 | 1.00 (Ref.) | 1.00 (Ref.) | 1.00 (Ref.) |
| Q2 | 2.0 (1.2-3.3) ** | 2.0 (1.2-3.4) ** | 1.9 (1.0-3.7) |
| Q3 | 3.3 (2.0-5.5) ** | 3.3 (2.0-5.6) ** | 2.0 (1.1-3.9) * |
| Q4 | 6.4 (4.1-10.0) ** | 6.7 (4.3-10.4) ** | 3.1 (1.7-5.6) ** |
| TC< 5.2 mmol/L |  |  |  |
| Q1 | 1.00 (Ref.) | 1.00 (Ref.) | 1.00 (Ref.) |
| Q2 | 1.5 (1.1-2.0) * | 1.5 (1.1-2.1) ** | 1.0 (0.6-1.5) |
| Q3 | 2.5 (1.7-3.5) ** | 2.6 (1.8-3.7) ** | 1.5 (0.9-2.5) |
| Q4 | 4.7 (3.1-7.0) ** | 5.0 (3.3-7.4) ** | 1.5 (0.9-2.5) |

1 Calculated using binary logistic regression.

Model 1 adjusted for race.

Model 2 adjusted for race, body mass index, waist circumference, drinking status, education level, hypertension, serum triglyceride, total cholesterol, urate lowering therapy.

* *P* < 0.05

** *P* < 0.01
